# Ultrafast nonlinear optical response of Dirac fermions in graphene

**DOI:** 10.1038/s41467-018-03413-7

**Published:** 2018-03-09

**Authors:** Matthias Baudisch, Andrea Marini, Joel D. Cox, Tony Zhu, Francisco Silva, Stephan Teichmann, Mathieu Massicotte, Frank Koppens, Leonid S. Levitov, F. Javier García de Abajo, Jens Biegert

**Affiliations:** 1 ICFO-Institut de Ciencies Fotoniques, The Barcelona Institute of Science and Technology, 08860 Barcelona, Spain; 2 Department of Physics, Massachusetts Institute of Technology, Cambridge, MA 02139 USA; 3 ICREA, Pg. Lluís Companys 23, 08010 Barcelona, Spain; 4 Present Address: Department of Physical and Chemical Sciences, University of L’Aquila, Via Vetoio 10, I-67100 L’Aquila, Italy

## Abstract

The speed of solid-state electronic devices, determined by the temporal dynamics of charge carriers, could potentially reach unprecedented petahertz frequencies through direct manipulation by optical fields, consisting in a million-fold increase from state-of-the-art technology. In graphene, charge carrier manipulation is facilitated by exceptionally strong coupling to optical fields, from which stems an important back-action of photoexcited carriers. Here we investigate the instantaneous response of graphene to ultrafast optical fields, elucidating the role of hot carriers on sub-100 fs timescales. The measured nonlinear response and its dependence on interaction time and field polarization reveal the back-action of hot carriers over timescales commensurate with the optical field. An intuitive picture is given for the carrier trajectories in response to the optical-field polarization state. We note that the peculiar interplay between optical fields and charge carriers in graphene may also apply to surface states in topological insulators with similar Dirac cone dispersion relations.

## Abstract (web summary)

Graphene possesses a nonlinear optical response arising from its electronic dispersion. Here, the authors measure the response of graphene to an ultrafast optical field and provide an explanation of the quantum dynamics of Dirac carriers mediating the material’s nonlinear response.

## Introduction

Graphene is a remarkable material^1^ that exhibits fascinating properties, such as ultrahigh electron mobility^2,3^, large mechanical strength^4^, and extraordinary optoelectronic behavior^5–7^. In particular, its electron transport properties^8,9^, which are described by a two-dimensional gas of massless Dirac fermions (MDFs) moving at a velocity of 1 nm fs^−1^, are of interest to develop ultrafast optoelectronic devices^10^. Its room-temperature carrier mobility of up to 1.5×10^6^ cm^2^ V^−1^ s^−1^ is unusually high^2^, while the third-order susceptibility of a single-atom-thick graphene layer is 10^8^ times stronger than in a dielectric material^11^. The large nonlinear response of graphene^11–14^ is attributed to its linear electronic energy dispersion^17^, which not only provides resonant interband transitions for a continuous range of low photon energies, but in the presence of intense optical fields leads to square-wave oscillatory motion of Dirac fermions (i.e., an anharmonic current response of the material and the emission of new frequencies of light). Third-harmonic generation^15,16^ and four-wave mixing were observed early on, while an extreme nonlinear effect has been recently reported, consisting of transient plasmons enabled by strong optical pumping^17^. High-harmonic generation (HHG) up to the 5^th^ order has been also observed at THz frequencies^18^, and more recently the light polarization dependence has been investigated for high harmonics^19^. Additionally, the introduction of a slight bandgap in the graphene dispersion effectively enhances the nonlinear response compared to that of gapless graphene^20,21^. Consequently, the nontrivial interactions among optical fields and Dirac fermions in graphene, even at low-field strengths, demands an investigation of the non-equilibrium response of the material’s charge carriers as a first step toward the implementation of ultrafast optoelectronic control^10,22,23^.

Here, we focus on the ultrafast nonlinear response of graphene arising from the photo-generated free carriers at moderate pump intensity, describing their effect on HHG along with the dependence on elliptical polarization, which we find quite different from ref. ^19^. Due to the low-field regime of our investigation. In particular, we observe a blue-shifted nonlinear response that is not unique to graphene and is known to occur in semiconductors (e.g., silicon^24,25^), as well as in gas-filled photonic crystal fibers undergoing ionization^26^. Our findings reveal that remarkably, despite the negligible propagation path length within the two-dimensional medium, the blue-shift signature is measurable for the generated harmonics, thus providing insight into the ultrafast dynamics of MDFs in graphene, matched by excellent agreement with simulations.

## Results

### Non-equilibrium carrier dynamics of grapheme

**Fig. 1:**
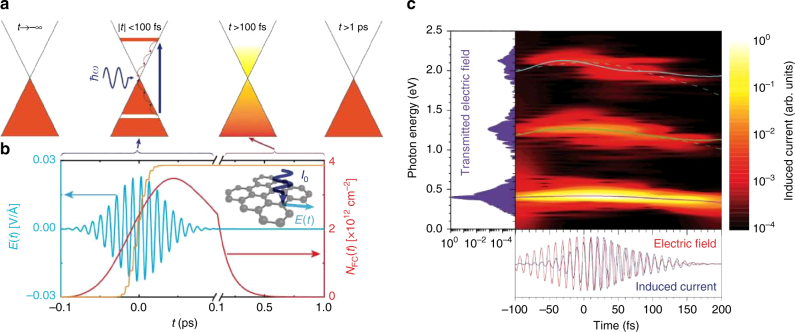
Ultrafast response and dynamics of Dirac fermions. **a** Schematic representation of the ultrafast temporal dynamics of photoexcited electrons in extended graphene. **b** Temporal evolution of the free carrier density *N*_FC_ (red curve, right vertical axis) generated by a normally-impinging linearly polarized Gaussian optical pulse (cyan curve, left axis) of 7 GW cm^−2^ peak intensity and 50 fs FWHM duration, as obtained from the MDF model (see Methods) for monolayer graphene. A characteristic ionized fraction of an atomic gas target is shown for comparison as calculated with PPT theory^27^ (orange curve). **c** Gabor analysis (150 fs Gaussian time window) of the squared induced current (color plot), along with the spectral (left plot) and temporal (lower plot) dependences of the transmitted electric field. Solid curves superimposed onto the color plot correspond to the time-dependent spectral centroid of the fundamental and harmonic intensities, while the two dashed curves are obtained by multiplying the energy of the fundamental centroid by factors of 3 and 5

Figure 1a illustrates the temporal dynamics of graphene electrons upon intense optical pulse irradiation over various timescales. In contrast to other materials, the conical band structure of graphene facilitates the excitation of electron-hole pairs at any instant of time during irradiation with an optical field, thus resulting in a near-instantaneous generation of non-thermal free carriers with metal-like optical response. This is followed by electronic thermalization due to electron–electron collisions over a characteristic time scale of ~50 fs^28,29^. Finally, electron–phonon coupling leads to electron relaxation and electron-hole recombination, thereby gradually reducing the electron temperature over a characteristic picosecond time scale. The described non-equilibrium carrier dynamics of graphene have been experimentally investigated, in particular through measurements in the THz regime^18,28,30^ and also using ca. 10-fs-duration pulses in the visible^29^. Such measurements were instrumental to elucidate the dynamics of charge carriers in this material, but an implementation of ultrafast optoelectronics requires additional knowledge on how charge carriers influence the optical fields themselves.

### Optical stimulus and response of grapheme

Here, we investigate the direct back-action of the carrier dynamics on the optical field at the petahertz scale through harmonic generation. Indeed, spectral reshaping of the fundamental optical field is amplified by the harmonic order, and the response at the generated frequencies is disentangled from that of the fundamental excitation pulse itself. We employ two different theoretical models to describe this interaction (see Supplementary Materials): one based on a non-perturbative continuum picture in which *π* electrons are treated as MDFs; and complementary time-domain simulations combining an atomistic tight-binding model for the electrons with the random-phase approximation formalism (TB-RPA). We find that these two approaches yield results in excellent agreement for the experimentally relevant conditions.

Figure 1b shows the predicted temporal evolution of free carriers (red curve) in response to the optical field (blue curve) of an experimental pulse with 70-fs FWHM duration at a photon energy of 0.4 eV and with peak intensity of 5 GW cm^−2^. The absence of a bandgap, in combination with the linear energy dispersion of graphene, leads to an instantaneous photo-generation of free carriers by the optical field and a delayed recombination with a metal-like instantaneous response and time-dependent plasma frequency. This behavior is markedly different compared to systems with a large bandgap, such as free atoms and insulators^10,31,32^. To illustrate the difference, we show the temporal evolution of the ionization yield for an atomic medium (orange curve), where the well-known step-like response of the emitted electrons is clearly discernable. Depending on the strength of the optical field, the medium may be fully ionized already at the leading edge of the pulse, in such a way that the trailing edge experiences a constant carrier density. The scenario is distinctly different in graphene because free carriers are instantaneously generated whenever an optical field is present. In addition, efficient and rapid electron–electron scattering occurs due to the high mobility of free carriers, thus affecting the optical field in a transient manner, which is markedly different from the abrupt temporal variation of the atomic medium.

Consequently, in graphene the generated free carriers oscillate synchronously with the optical field at early times (leading edge of the pulse), but modify the optical response for later times during the optical field (trailing part of the pulse). The result is an induced asymmetry on the leading and trailing parts of the optical field (Fig. 1b), and a switchover from dielectric- to metallic-like material response along the evolution of the optical field. The bottom panel in Fig. 1c shows the resulting temporal response of the simulated induced current, which confirms that free carriers oscillate synchronously with the optical field at early times, but later acquire a phase lag due to the increasing density of free carriers.

The back-action of charge carrier motion to the optical field is revealed by the time-energy plot of the resulting nonlinear light emission in Fig. 1c. New optical frequencies, at odd orders of the fundamental (left panel), are generated due to the anharmonic response from the optical-field-driven square-wave oscillatory motion of Dirac fermions. Additionally, the free carriers lower the refractive index and cause a spectral blue-shift that is proportional to the optical frequency (main panel). Because of this interplay, the optical pulse accelerates at the trailing edge, while the pulse envelope undergoes self-steepening at the leading front.

### Measurement of the polarization response and harmonic generation

**Fig. 2:**
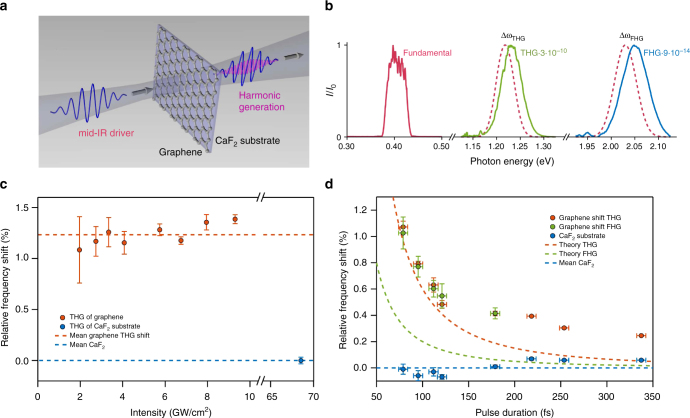
Blue-shifted harmonics. **a** Schematic of the experimental configuration, showing the mid-IR field (linearly polarized, 70-fs pulses at 3.1 μm wavelength, i.e., *E*_0_ = 0.4 eV photon energy) before and after propagation through 5 monolayers of graphene that are supported on a 0.4-mm thick CaF_2_ substrate. Pulses of different durations and degrees of elliptical polarization are also investigated. **b** Measured fundamental spectrum, along with the emission at the third and fifth harmonics (blue-shifted). The dashed curves represent the nominal position of the third and fifth harmonic. **c** The resulting third harmonic is blue-shifted by 1.8% from 3*E*_0_ and the blue-shift is independent of driving field intensity. **d** The blue-shift depends inversely on pulse duration for both the third and the fifth harmonic. Note that, while in (**b**) we plot the absolute spectral shift, in (**d**) we plot the relative shift δω_THG_/3ω and δω_FHG_/5ω. The measurement errors are derived according to the error propagation law from the instrument measurement uncertainties, i.e., from the spectrometer, power meter, beam profiler, and frequency resolved optical gating

Experimentally, direct observation of back-action in the temporal domain requires petahertz-level sub-cycle sampling of the optical field, but the effect is clearly revealed in the spectral domain through blue-shifts and the generation of new optical frequencies. For our experiment, we used 70-fs (6.4 optical cycles) FWHM pulses with a central wavelength of 3.1 μm (0.4 eV photon energy) from a mid-IR optical parametric chirped pulse amplifier (OPCPA)^33^. We focused the pulses at normal incidence onto a 5-monolayer graphene sample that was transferred onto a 0.4-mm thick CaF_2_ substrate (see Fig. 2a and Supplementary Materials). Reference measurements were performed on the CaF_2_ substrate without graphene to exclude any additional contribution for peak intensities below 10 GW cm^−2^. Graphene irradiation at higher peak intensity resulted in an irreversibly decreasing signal over time, indicating material damage. The transmitted mid-IR field and the generated new optical frequencies were collected with a spectrometer.

A characteristic measurement for linear polarization is shown in Fig. 2b for irradiation at a peak intensity of 5 GW cm^−2^. We observed the generation of harmonic frequencies up to fifth order and with proportional blue-shifts compared to the nominal third and fifth harmonic (dashed curves). This first observation of the fifth harmonic is commensurate with predictions^12,13,34,35^ and contradicts earlier measurements in multilayer graphene at terahertz frequencies in which the absence of optical harmonics was attributed to fast carrier scattering^36^, which at THz frequencies is heavily affected by hot thermal carriers. To further investigate the influence of the charge carriers, we measured the scaling of the blue-shift with peak intensity and pulse duration. Longer pulses, up to 340 fs duration, at constant peak intensity, were generated by spectrally narrowing the laser output and increasing the pulse energy proportionally with the increase of pulse duration. We resorted to spectral narrowing rather than simple frequency chirping to exclude additional dynamical effects associated with pulse propagation in graphene. Figure 2c shows that the measured blue-shifts did not depend on peak intensity for constant pulse duration. By fixing the peak intensity and changing the duration of the pulse, we observed, however, that the blue-shifts of the third and fifth harmonics scaled inversely with pulse duration. These observations are in excellent qualitative agreement with theoretical simulations, which are overlaid in Fig. 2c and d (dashed curves). Note that the measured blue-shift of the fifth harmonic does not show a quantitative agreement as good as the third harmonic, although qualitatively similar. This may be due to several experimental effects not accounted for by the theory (e.g., the chromatic dispersion of the substrate, the spectrally inhomogeneous absorption due to the substrate, the presence of defects in the graphene structure, and the shape of the input pump pulse, assumed to be a Gaussian in the numerical simulations, but actually quite deformed and chirped in the experiments). Because measurements are performed at an impinging photon energy of 0.4 eV, the interband dynamics is very weakly affected by small energy bandgaps induced by multilayers or intrinsic doping of the order of 0.1 eV. The induced blue-shift is an interband effect, and, thus, is not strongly sensitive on small modifications of the material structure, in particular the small energy bandgap induced by the multilayer.

### Quantum dynamics of Dirac carriers

**Fig. 3:**
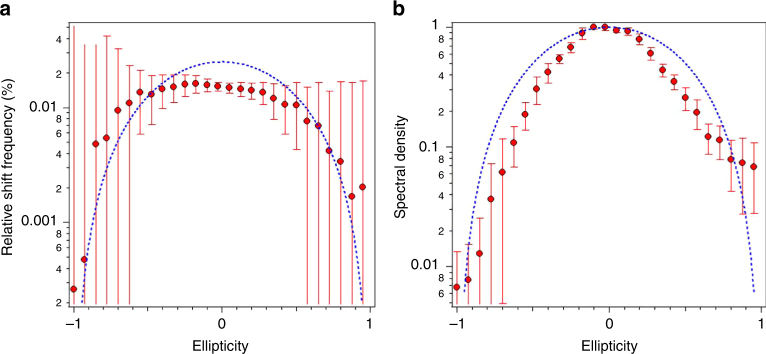
Polarization dependence. We show the dependence of the third harmonic blue-shift (**a**) and intensity (**b**) as a function of ellipticity of the impinging light. Measurements (symbols with error bars) are compared with MDF simulations (solid curves). The measurement errors are derived from the instrument measurement uncertainties, i.e., from the spectrometer and power meter

To gain further insight into carrier dynamics in *k*-space and their effect on the nonlinear response, we repeated our measurements with varying polarization for the incident mid-IR field. Figure 3a shows that the third harmonic signal strength decreased rapidly with increasing ellipticity *∈* and that the resulting third harmonic was two orders of magnitude weaker for a circularly polarized field (*ε* = 1) compared with a linearly polarized field (*ε* = 0). Of particular interest is the dependence of the blue-shift on ellipticity^37^. Fig. 3b shows that the blue-shift was maximal (~2% of the central frequency) for linear fields and decreased to values lower than 0.3% for circularly polarized light.

**Fig. 4:**
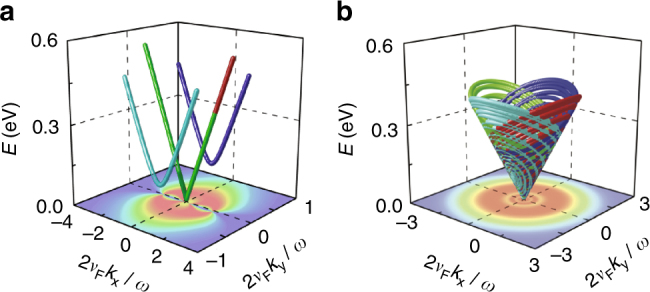
Dirac fermion evolution in *k*-space. We show trajectories for linear (**a**) and circularly polarized (**b**) incident light and different initial *k*-space conditions (plotted with different colors, see Supplementary Materials). The out-of-equilibrium *k*-space density of free carriers is shown in the lower color plot

Our simulations qualitatively reproduce the experimental findings and are indicated by the dashed blue curves superimposed on the experimental data presented in Fig. 3. It should be noted that, while the reduction of generated free carriers in atoms and molecules due to the scaling of ionization and decrease in refractive index^38^ with circular polarization are well-known phenomena, the scenario here investigated is markedly different due to the availability of carriers, the ballistic transport of Dirac fermions, and the band topology in graphene. Our simulations clearly confirm a reduction of anharmonic response, and hence a diminished harmonic generation that reproduces the measured reduction in blue-shift with increasing ellipticity. The origin of this behavior becomes clear from a trajectory analysis of field-driven Dirac fermions, as shown in Fig. 4 for the extreme cases of linear and circularly polarized driving fields (see Supplementary Figs. 1 and 2 for additional studies of the polarization dependence). With linear polarization, low-energy carriers are field-driven across the anharmonic potential of the Dirac cone (Fig. 4a) and the emission of harmonics is the direct consequence of such anharmonic motion. Along their excursion in the Dirac potential, there is a large probability that the charge carriers re-encounter the lowest energy point of the Dirac cone, where the dipole matrix element diverges, thus enhancing the generation of free carriers as well as the consequential blue-shift. The dynamics are very different when graphene is driven by circularly polarized light (Fig. 4b), for which charge carriers follow spiraling trajectories along the Dirac cone. In contrast to the linear driving scenario, the carriers do not encounter the lowest energy point twice during each field cycle, but instead only at the starting and ending parts of the pulse. More precisely, under the action of a circularly polarized pulse, carriers are driven in spiraling trajectories ascending the Dirac cone with increasing field amplitude and back down with diminishing field amplitude, thus acquiring a 2*π* phase in *k*-space during each optical cycle. The availability of carriers across *k*-space ensures that a matching trajectory always exists that is *π* out of phase, so that any anharmonic response is effectively canceled for circular driving fields. It is worth emphasizing that the observed ellipticity dependence of harmonic generation is not unique to graphene because it inherently arises from crystal symmetry. Three-fold rotational symmetry (which is present in graphene) generally forbids THG under circularly polarized illumination^39^. Thus, in a simple picture, the quenching of blue-shift for circularly polarized light can be readily understood from the vanishing of the harmonic generation process.

## Discussion

We identify both the dynamics of graphene Dirac carriers driven by optical petahertz fields and the back-action of these dynamics on the driving field as revealed by blue-shifts in the generation of third and fifth harmonics. Theoretical modeling explains the results and allows us to track down carrier trajectories in the specific topology of Dirac cones, which readily explains the disappearance of the blue-shifts when the incident light is circularly polarized. These investigations of the dynamics of Dirac carriers in graphene provide important insight for the design of future graphene-based ultrafast optoelectronic devices, while they open an avenue toward the exploration of topology-dependent carrier dynamics in other two-dimensional systems, such as surface states of topological insulators and other two-dimensional materials.

## Methods

### Additional experimental information

The graphene sample consists of a 5 large (~1 cm^2^) graphene monolayers grown by chemical vapor deposition on a sputtered Cu film (Aixtron) and transferred on top of one another onto a 0.4-mm thick CaF_2_ substrate. Each graphene layer is transferred by spin-coating polymethyl methacrylate (PMMA) on top of the graphene/Cu surface and etching the Cu copper using an oxidizing solution of 0.1 M ammonium persulfate. The floating PMMA/graphene layer is transferred to DI water and scooped using the CaF_2_ substrate. Once the sample is dry, PMMA is removed with acetone and the sample is rinsed in isopropanol. We performed electrical measurements on similar graphene samples and typically found an intrinsic hole doping of ~5 x 10^12^ cm^−2^, with a room-temperature hole mobility of ~500 cm^2^ V^−1^ s^−1^.

In the experiment, the mid-IR output of an OPCPA system is used as fundamental pump beam to investigate the optical response of graphene. We used output pulses at a center wavelength of 3.1 μm, with pulse energies of 1.3 μJ and pulse durations of 80 fs at a pulse repetition rate of 160 kHz. In the experimental setup, the mid-IR driving pulses are focused with a 30-cm-focal-length CaF_2_ lens onto the graphene sample resulting in FWHM beam sizes between 50 and 200 μm. The driving pulse energy was set by a combination of half-wave plate and polarizer and the pulse duration was tuned by narrowing the spectrum and compensating with energy to achieve identical peak intensity. Beyond peak intensities of 10 GW cm^−2^ we observed that the third harmonic signal from graphene decreased irreversibly with time suggesting light- induced damage to the sample. Interestingly enough, the damage did not affect the center wavelength of the generated third harmonic. The reference measurement with CaF_2_ was done by placing the plate into focus to increase signal strength to a level comparable to the graphene sample. This corresponded to peak intensities of up to 2.8 TW cm^−2^. Ellipticity variations of the polarization of the fundamental pulses were realized with a combination of a *λ*/2-plate and a *λ*/4-plate (B. Halle). The electric field components along the major and minor axes of the polarization ellipse were separated with a polarizing beam splitter cube (B. Halle) placed after the sample. The generated third harmonic signal was separated from the fundamental and simultaneously focused into a fiber-coupled spectrometer (HR4000, Ocean Optics) with transmissive BK7 optics.

### Theoretical modeling

Our simulations are based on two complementary approaches to describe the nonlinear optical response of graphene: (i) a non-perturbative continuum picture in which *π* electrons are treated as MDFs; and (ii) an atomistic tight-binding model for the electrons combined with the TB-RPA. These two approaches yield results in excellent mutual agreement under the conditions of the present experiment. (i) The MDF model (see Supplementary Note 1) introduces light-matter interaction through the electron quasi-momentum **k** + (*e*/*c*)**A**, where **k** is the electron momentum, whereas ${\bf{A}}(t) = - c{\int}_{ -\infty}^t {{\bf{E}}\left( {t\prime } \right){\rm d}t\prime }$ is obtained from the incident light electric field **E**. Electrons are treated independently and their dynamics described by the Dirac equation for massless fermions, which can be recast as in the form of Bloch equations, involving the population inversion *n*_**k**_(**R**, *t*), the interband coherence *ρ*_**k**_(**R**, *t*), and a phenomenological relaxation time *τ*. We numerically integrate these equations to extract the **k**-resolved induced current and is integral over **k** to yield the total current. A self-contained presentation of this method is given in Supplementary Information (see Supplementary Note 1), along with analytical expressions for the **k-**space resolved contribution to harmonic generation under CW illumination (see Supplementary Note 2). Pulses are also simulated by direct time integration of the Bloch equations. (ii) In the TB-RPA method (see Supplementary Note 3), we solve the one-particle density matrix $\dot \rho = - \left( {\frac{i}{h}} \right)\left[ {H_{\rm TB} - e\phi ,\rho } \right] - \left( {\frac{1}{{2\tau }}} \right)\left( {\rho - \rho ^ \circ } \right)$ to describe the temporal dynamics of graphene electrons, where *H*_TB_ is the nearest-neighbors tight-binding Hamiltonian (2.8 eV hopping energy, one orbital per carbon site) and *ϕ* is the electric potential produced by the incident light plus the Hartree interaction. We express the density matrix $\rho = \mathop {\sum}\nolimits_{jj{\prime}} {\left| j \right\rangle \left\langle {j\prime } \right|}$ in terms of the one-electron eigenstates of *H*_TB_ with complex-number expansion coefficients $\rho _{jj\prime }$. The relaxed state of the system is $\rho _{jj{\prime}}^ \circ = \delta _{jj{\prime }}$ for occupied states and 0 otherwise. The spectrally resolved induced field is then readily obtained by Fourier transforming the $\rho _{jj\prime }$ coefficients. Calculations are performed for undoped ribbons of increasing width until convergence is achieved. We assume a relaxation time *τ* = 100 fs in both approaches.

### Data availability

The data that support the findings of this study are available from the corresponding author upon request.

## Electronic supplementary material

Supplementary Information
