# Supplementary material for: Ultrafast nonlinear optical response of Dirac fermions in graphene
Source: Nat Commun. 2018 Mar 9;9:1018. doi: 10.1038/s41467-018-03413-7 (PMC5844892; doi:10.1038/s41467-018-03413-7)
Supplement: Supplementary file 1 — Supplementary Information [file 41467_2018_3413_MOESM1_ESM.pdf]

Supplementary Materials for
Ultrafast nonlinear optical response of Dirac fermions in graphene

Matthias Baudisch,[1] Andrea Marini,[1,2] Joel D. Cox,[1] Tony Zhu,[3] Francisco Silva,[1] Stephan Teichmann,[1] Mathieu Massicotte,[1] Frank Koppens,[1,4] Leonid S. Levitov,[1,4] F. Javier García de Abajo,[1,4] and Jens Biegert*[1,4]

[1]*ICFO-Institut de Ciencies Fotoniques, The Barcelona Institute of Science and Technology, 08860 Castelldefels (Barcelona), Spain*
[2]*Current address: Department of Physical and Chemical Sciences, University of L'Aquila, via Vetoio 10, I-67100 L'Aquila, Italy*
[3]*Department of Physics, Massachusetts Institute of Technology, Cambridge, MA 02139, USA*
[4]*ICREA-Institució Catalana de Recerca i Estudis Avançats, Barcelona, Spain*

Supplementary Figures

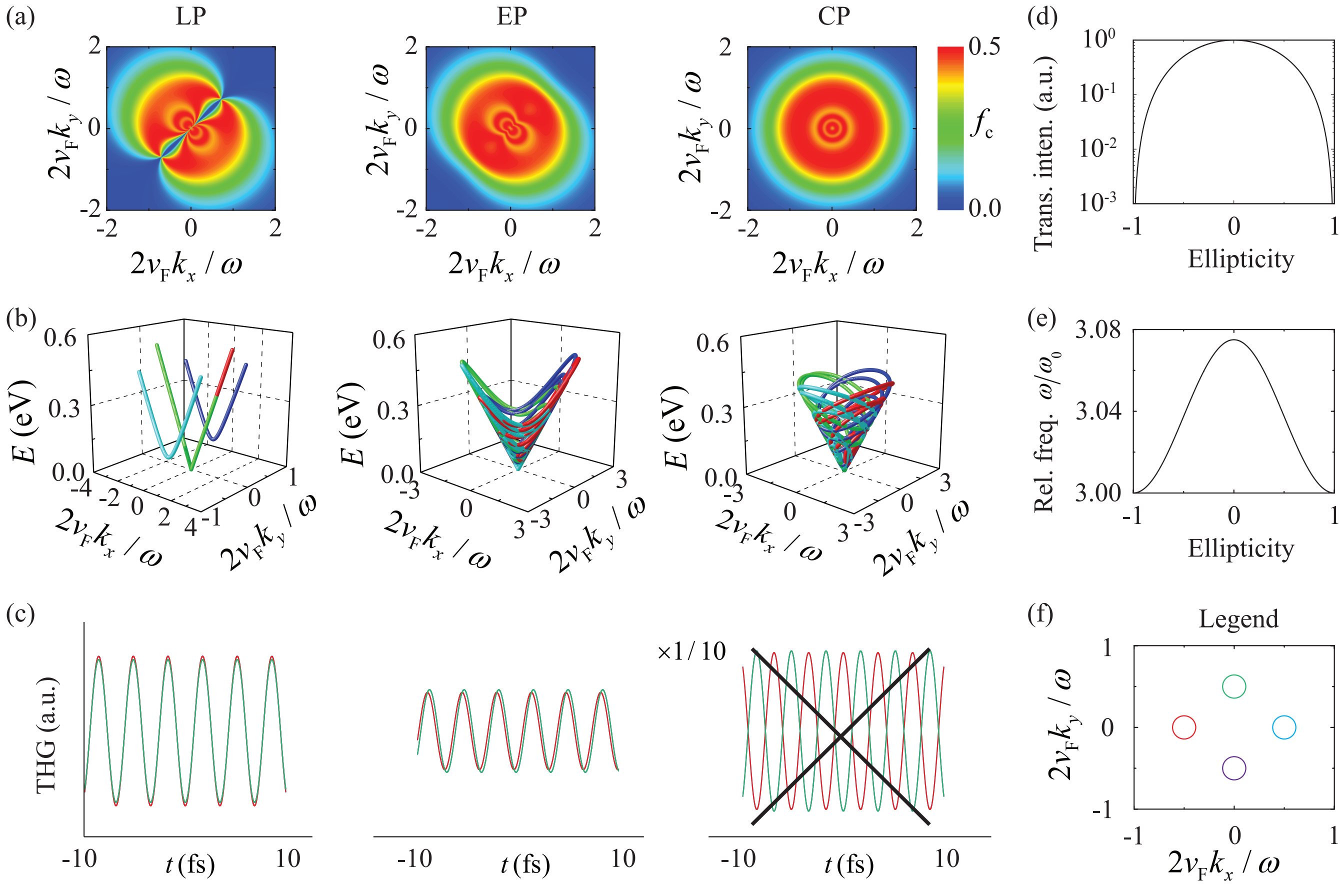

Supplementary Figure 1: Polarization dependence of (a) photoexcited free-carriers, (b) electron trajectories in $\mathbf{k}$ space, (c) third-harmonic currents from different electrons, (d) transmitted third-harmonic intensity, and (e) corresponding blueshift. Different colors of curves in (b,c) correspond to different points in $\mathbf{k}$ space illustrated in (f). Plots (a,c) illustrate the analytical derivation of (a) out-of-equilibrium carriers and (c) currents in Sec. II, while plots (b,c,d) are achieved though numerical integration of the full GBEs [Sec. I, Supplementary Equations (1,2)]. Labels LP, EP, and CP indicate linear ($\vartheta = 0$), elliptical ($\vartheta = \pi/4$), and circular polarization ($\vartheta = \pi/2$), respectively.

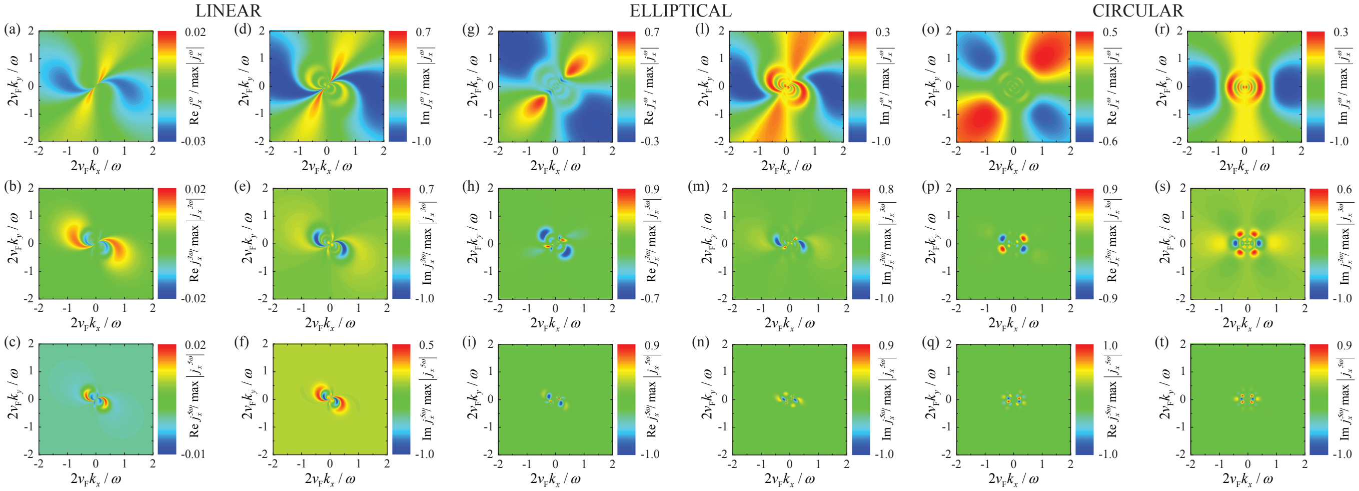

Supplementary Figure 2: Dependence of (a,g,o) $\mathrm{Re}j_x^{\omega}/\mathrm{max}|j_x^{\omega}|$, (d,l,r) $\mathrm{Im}j_x^{\omega}/\mathrm{max}|j_x^{\omega}|$, (b,h,p) $\mathrm{Re}j_x^{3\omega}/\mathrm{max}|j_x^{3\omega}|$, (e,m,s) $\mathrm{Im}j_x^{3\omega}/\mathrm{max}|j_x^{3\omega}|$, (c,i,q) $\mathrm{Re}j_x^{5\omega}/\mathrm{max}|j_x^{5\omega}|$, and (f,n,t) $\mathrm{Im}j_x^{5\omega}/\mathrm{max}|j_x^{5\omega}|$ over the dimensionless electron wavenumber $2v_{\rm F}\mathbf{k}/\omega$ for wavelength $\lambda = 2\pi c/\omega = 3.1\ \mu$m, impinging intensity $I_0 = 10$ GW/cm^2, and several polarization states: (a-f) linear $\vartheta = 0$, (g-n) elliptical $\vartheta = \pi/4$, and (o-t) circular $\vartheta = \pi/2$.

Supplementary Note 1: Extended graphene in the Massless Dirac fermion picture

We consider an optical field $\mathbf{E}(t) = \mathbf{E}_0(t)\mathrm{e}^{-i\omega t} + \mathrm{c.c.}$ of carrier angular frequency ω and complex envelope amplitude $\mathbf{E}_0(t)$ impinging at normal incidence on a self-standing extended graphene sheet. At visible and lower frequencies, electrons in this material behave as massless Dirac fermions (MDFs), with their temporal evolution governed by the single-particle Dirac equation $i\hbar\partial_t\psi_{\mathbf{k}}(t) = v_{\rm F}\boldsymbol{\pi}\cdot\boldsymbol{\sigma}\psi_{\mathbf{k}}(t)$, where $\hbar\mathbf{k}$ is the electron momentum, ∂_t is the time derivative, $v_{\rm F} \simeq c/300$ is the Fermi velocity, c is the speed of light in vacuum, $\hbar$ is the reduced Planck constant, $\boldsymbol{\sigma} = (\sigma_x,\ \sigma_y)$ is the two-dimensional (2D) Pauli-matrix vector, and $\psi_{\mathbf{k}}(t)$ is the $\mathbf{k}$- and time-dependent two-component spinor accounting for the quantum states in the upper and lower Dirac-cone bands. The graphene band-structure thus consists of two infinite cones, neglecting higher-band effects that are only relevant at high photon energies, above $\sim 2\,$eV. We introduce an electron quasi-momentum $\boldsymbol{\pi}$ that coincides with the unperturbed momentum (i.e., $\boldsymbol{\pi} = \hbar\mathbf{k}$) in the absence of external illumination. In this case, the Dirac equation admits spinor eigenvectors $\psi^{\pm}_{\mathbf{k},0}(t) = (1/\sqrt{2})(\mathrm{e}^{-i\phi/2}\quad \pm \mathrm{e}^{i\phi/2})^{\rm T}\mathrm{e}^{-i\varepsilon_{\pm}t}$, where $\varepsilon_{\pm} = \pm v_{\rm F}k$ is the unperturbed conical dispersion of upper $(+)$ and lower $(-)$ energy bands, while ϕ identifies the momentum direction, such that $\mathbf{k} = k(\cos\phi, \sin\phi)$, and a spatial dependence $\mathrm{e}^{i\mathbf{k}\cdot\mathbf{r}}/\sqrt{\mathcal{A}}$ (normalized to the sheet area $\mathcal{A}$) is understood in the spinor.

In the presence of the impinging optical field, we use the customary minimal electron-light coupling prescription to write the electron quasi-momentum as $\boldsymbol{\pi}(t) = \hbar\mathbf{k} + (e/c)\mathbf{A}(t)$, where $-e$ is the electron charge and $\mathbf{A}(t) = -c\int\mathbf{E}(t)dt$ is the potential vector in the Coulomb gauge $(\nabla\cdot\mathbf{A} = 0)$. Following the nonperturbative approach developed by Ishikawa[1,2], we write the time-dependent spinor as a linear combination of the instantaneous upper- and lower-cone states $\psi_{\mathbf{k}}(t) = c^{+}_{\mathbf{k}}(t)\psi^{+}_{\mathbf{k}}(t) + c^{-}_{\mathbf{k}}(t)\psi^{-}_{\mathbf{k}}(t)$, where $\psi^{\pm}_{\mathbf{k}}(t) = (1/\sqrt{2})(\mathrm{e}^{-i\theta_{\mathbf{k}}(t)/2}\quad \pm \mathrm{e}^{i\theta_{\mathbf{k}}(t)/2})^{\rm T}\mathrm{e}^{\mp i\Omega_{\mathbf{k}}(t)}$, $\Omega_{\mathbf{k}}(t) = v_{\rm F}\int|\mathbf{k} + (e/\hbar c)\mathbf{A}(t)|\,dt$ is a global dynamical phase, and $\theta_{\mathbf{k}}(t) = \mathrm{atan}\{[k_y + (e/\hbar c)A_y(t)]/[k_x + (e/\hbar c)A_x(t)]\}$ is the time-dependent direction angle of the electron quasi-momentum $\boldsymbol{\pi}$. We then insert the Ansatz above into the Dirac equation and define the interband coherence $\rho_{\mathbf{k}} = c^{+}_{\mathbf{k}}c^{-*}_{\mathbf{k}}$ and the population difference $n_{\mathbf{k}} = |c^{+}_{\mathbf{k}}|^2 - |c^{-}_{\mathbf{k}}|^2$. Without making any approximations, we can then rewrite the two-dimensional Dirac equation for MDFs in the form of generalized Bloch equations (GBEs)[1,2],

$$\dot{\rho}_{\mathbf{k}}(t) = -\gamma\rho_{\mathbf{k}}(t) - \frac{i}{2}\dot{\theta}_{\mathbf{k}}(t)n_{\mathbf{k}}(t)\mathrm{e}^{2i\Omega_{\mathbf{k}}(t)}, \tag{1}$$

$$\dot{n}_{\mathbf{k}}(t) = -\gamma[n_{\mathbf{k}}(t) + 1] + 2\dot{\theta}_{\mathbf{k}}(t)\mathrm{Im}\left\{\rho_{\mathbf{k}}(t)\mathrm{e}^{-2i\Omega_{\mathbf{k}}(t)}\right\}, \tag{2}$$

where we have introduced the artificial damping parameter γ accounting for inelastic electron transitions that are produced for example by impurity scattering and coupling to phonons. The temporal evolution of graphene electrons can be evaluated by solving the above equations in the time-domain and setting the initial conditions $\rho_{\mathbf{k}}(-\infty) = 0$ and $n_{\mathbf{k}}(-\infty) = -1$, where we have assumed the limit of vanishing temperature and doping. The single-electron current is

thus obtained as

$$\mathbf{j_k}(t) = (-e/\hbar)\langle\psi_\mathbf{k}(t)|\nabla_\mathbf{k}[v_\mathrm{F}\boldsymbol{\pi}\cdot\boldsymbol{\sigma}]|\psi_\mathbf{k}(t)\rangle = -ev_\mathrm{F}\left[n_\mathbf{k}(\cos\theta_\mathbf{k}\hat{\mathbf{x}}+\sin\theta_\mathbf{k}\hat{\mathbf{y}}) - 2(\sin\theta_\mathbf{k}\hat{\mathbf{x}}-\cos\theta_\mathbf{k}\hat{\mathbf{y}})\mathrm{Im}\left\{\rho_\mathbf{k}\mathrm{e}^{-2i\Omega_\mathbf{k}}\right\}\right]. \quad (3)$$

We emphasize that this expression for the microscopic current is obtained without making any approximation beyond the MDF picture. The $n_\mathbf{k}$ terms account for the intraband current, while the remaining terms, which depend on the coherence $\rho_\mathbf{k}$, arise from interband dynamics[1,2]. One should note that the intraband current of the valence band $n_\mathbf{k}\cos\theta_\mathbf{k}$ should vanish when it is fully filled ($n_\mathbf{k} = -1$) in virtue of a well-known sum rule (see[3]). The macroscopic induced surface current $\mathbf{J}(t)$ is finally obtained by integrating over all electron momenta,

$$\mathbf{J}(t) = \frac{g_\mathrm{s}g_\mathrm{v}}{(2\pi)^2}\int \mathbf{j_k}(t)d^2\mathbf{k}, \quad (4)$$

where $g_\mathrm{s} = g_\mathrm{v} = 2$ account for spin and valley degeneracies, and the integral extends over the entire 2D $\mathbf{k}$ plane. It is important to note that this integral is ill-behaved due to the unphysical assumption of an infinitely extended valence band (i.e., for arbitrarily large k's). The noted sum rule explains that this divergence must disappear in real systems due to the periodicity of the band in $\mathbf{k}$ space. A regularization procedure to tackle this problem leads to the replacement of $n_\mathbf{k}$ in Supplementary Equation (3) with $n_\mathbf{k}+1$ when calculating the integral of Supplementary Equation (4), and this prevents the divergence since $n_\mathbf{k}\to -1$ for large k's[4]. In order to calculate the surface current density $\mathbf{J}(t)$ induced on graphene by the impinging light we solve numerically Supplementary Equations (1,2) through a fourth-order Runge Kutta algorithm. The transmitted field $\mathbf{E}_\mathrm{T}(t)$ is thus obtained by considering the scattering problem and applying the boundary conditions at the graphene plane accounting for the induced surface current density. As a result, at normal incidence we obtain $\mathbf{E}_\mathrm{T}(t) = \mathbf{E}(t) - (1/2)\mu_0 c\mathbf{J}(t)$. In the results given in the main paper, we considered a Gaussian input pulse $\mathbf{E}(t) = \mathbf{E}_0\mathrm{e}^{-(t/2\tau)^2 - i\omega t}$ + c.c. with several pulse durations τ, amplitudes E_0, and polarization states $\mathbf{E}_0$.

Supplementary Note 2: Continuous wave analysis of polarization-dependent third- and fifth- harmonic generation

Here, we provide an analytical demonstration of the quenching of harmonic generation for circularly polarized impinging light in the continuous-wave regime. We start our analysis from the Bloch equations under the approximation $\hbar k \gg (e/c)A(t)$. Such an approximation is valid in the regime where the optical intensity remains low enough not to temporally displace the Dirac cone up to the resonant electron wavenumber $k = \omega/(2v_\mathrm{F})$, leading to the (safely met) condition $I_0 << 137\hbar\pi^3c^4/2v_\mathrm{F}^2\lambda^4 \approx 2$ GW/cm^2 at $\lambda = 3.1$ μm. Under this approximation, the GBEs reduce to

$$\dot{\Gamma}_\mathbf{k} = -(2i\omega_0+\gamma)\Gamma_\mathbf{k} - \frac{ie}{2\hbar k}\left[\sin\phi E_x(t) - \cos\phi E_y(t)\right]\ n_\mathbf{k}, \quad (5)$$

$$\dot{n}_\mathbf{k} = -\gamma(n_\mathbf{k}+1) + \frac{2e}{\hbar k}\left[\sin\phi E_x(t) - \cos\phi E_y(t)\right]\ \mathrm{Im}\Gamma_\mathbf{k}, \quad (6)$$

where we have set $\rho_\mathbf{k} = \Gamma_\mathbf{k}\mathrm{e}^{2i\omega_0 t}$ and $\omega_0 = v_\mathrm{F}k$. Then, defining the polarization angle ϑ such that $E_x(t) = E_0\sin\omega t$ and $E_y(t) = E_0\sin(\omega t + \vartheta)$, and taking the Ansatz

$$\Gamma_\mathbf{k}(t) = \Gamma_\mathbf{k}^{(1+)}\mathrm{e}^{i\omega t} + \Gamma_\mathbf{k}^{(1-)}\mathrm{e}^{-i\omega t} + \Gamma_\mathbf{k}^{(3+)}\mathrm{e}^{3i\omega t} + \Gamma_\mathbf{k}^{(3-)}\mathrm{e}^{-3i\omega t} + \Gamma_\mathbf{k}^{(5+)}\mathrm{e}^{5i\omega t} + \Gamma_\mathbf{k}^{(5-)}\mathrm{e}^{-5i\omega t}, \quad (7a)$$

$$n_\mathbf{k}(t) = n_\mathbf{k}^{(0)} + \mathrm{Re}\left\{n_\mathbf{k}^{(2)}\mathrm{e}^{-2i\omega t}\right\} + \mathrm{Re}\left\{n_\mathbf{k}^{(4)}\mathrm{e}^{-4i\omega t}\right\} + \mathrm{Re}\left\{n_\mathbf{k}^{(6)}\mathrm{e}^{-6i\omega t}\right\}, \quad (7b)$$

one gets

$$\Gamma_{\mathbf{k}}^{(1-)} - \Gamma_{\mathbf{k}}^{(1+)*} = \frac{-eE_0(\gamma - i\omega)[\eta_{\mathbf{k}} n_{\mathbf{k}}^{(2)} - 2\eta_{\mathbf{k}}^* n_{\mathbf{k}}^{(0)}]}{4\hbar k[\gamma + i(2\omega_0 - \omega)][\gamma - i(2\omega_0 + \omega)]} \equiv \frac{\hbar k}{eE_0}\xi_1 \left[\eta_{\mathbf{k}} n_{\mathbf{k}}^{(2)} - 2\eta_{\mathbf{k}}^* n_{\mathbf{k}}^{(0)}\right], \quad (8a)$$

$$\Gamma_{\mathbf{k}}^{(3-)} - \Gamma_{\mathbf{k}}^{(3+)*} = \frac{-eE_0(\gamma - 3i\omega)[\eta_{\mathbf{k}} n_{\mathbf{k}}^{(4)} - \eta_{\mathbf{k}}^* n_{\mathbf{k}}^{(2)}]}{4\hbar k[\gamma + i(2\omega_0 - 3\omega)][\gamma - i(2\omega_0 + 3\omega)]} \equiv \frac{\hbar k}{eE_0}\xi_3 \left[\eta_{\mathbf{k}} n_{\mathbf{k}}^{(4)} - \eta_{\mathbf{k}}^* n_{\mathbf{k}}^{(2)}\right], \quad (8b)$$

$$\Gamma_{\mathbf{k}}^{(5-)} - \Gamma_{\mathbf{k}}^{(5+)*} = \frac{-eE_0(\gamma - 5i\omega)[\eta_{\mathbf{k}} n_{\mathbf{k}}^{(6)} - \eta_{\mathbf{k}}^* n_{\mathbf{k}}^{(4)}]}{4\hbar k[\gamma + i(2\omega_0 - 5\omega)][\gamma - i(2\omega_0 + 5\omega)]} \equiv \frac{\hbar k}{eE_0}\xi_5 \left[\eta_{\mathbf{k}} n_{\mathbf{k}}^{(6)} - \eta_{\mathbf{k}}^* n_{\mathbf{k}}^{(4)}\right], \quad (8c)$$

$$n_{\mathbf{k}}^{(0)} = -\gamma\left\{\gamma - \mathrm{Re}\left[2|\eta_{\mathbf{k}}|^2\xi_1 + \right.\right. \quad (8d)$$

$$\left.\left. + \frac{2|\eta_{\mathbf{k}}|^4\xi_1^2[(\gamma - 4i\omega - |\eta_{\mathbf{k}}|^2(\xi_3+\xi_5))(|\eta_{\mathbf{k}}|^2\xi_5 - \gamma + 6i\omega) + |\eta_{\mathbf{k}}|^4\xi_5^2]}{[\gamma - 2i\omega - |\eta_{\mathbf{k}}|^2(\xi_1+\xi_3)][(\gamma - 4i\omega - |\eta_{\mathbf{k}}|^2(\xi_3+\xi_5))(|\eta_{\mathbf{k}}|^2\xi_5 - \gamma + 6i\omega) + |\eta_{\mathbf{k}}|^4\xi_5^2] - |\eta_{\mathbf{k}}|^4\xi_3^2(|\eta_{\mathbf{k}}|^2\xi_5 - \gamma + 6i\omega)}\right]\right\}^{-1}, \quad (8e)$$

$$n_{\mathbf{k}}^{(2)} = \frac{-2(\eta_{\mathbf{k}}^*)^2\xi_1\{[\gamma - 4i\omega - |\eta_{\mathbf{k}}|^2(\xi_3+\xi_5)](|\eta_{\mathbf{k}}|^2\xi_5 - \gamma + 6i\omega) + |\eta_{\mathbf{k}}|^4\xi_5^2\} n_{\mathbf{k}}^{(0)}}{[\gamma - 2i\omega - |\eta_{\mathbf{k}}|^2(\xi_1+\xi_3)]\{[\gamma - 4i\omega - |\eta_{\mathbf{k}}|^2(\xi_3+\xi_5)](|\eta_{\mathbf{k}}|^2\xi_5 - \gamma + 6i\omega) + |\eta_{\mathbf{k}}|^4\xi_5^2\} - |\eta_{\mathbf{k}}|^4\xi_3^2(|\eta_{\mathbf{k}}|^2\xi_5 - \gamma + 6i\omega)}, \quad (8f)$$

$$n_{\mathbf{k}}^{(4)} = \frac{-(\eta_{\mathbf{k}}^*)^2\xi_3(|\eta_{\mathbf{k}}|^2\xi_5 - \gamma + 6i\omega) n_{\mathbf{k}}^{(2)}}{[\gamma - 4i\omega - |\eta_{\mathbf{k}}|^2(\xi_3+\xi_5)](|\eta_{\mathbf{k}}|^2\xi_5 - \gamma + 6i\omega) + |\eta_{\mathbf{k}}|^4\xi_5^2}, \quad (8g)$$

where we have introduced the dimensionless parameter $\eta_{\mathbf{k}} = \sin\phi - \cos\phi \mathrm{e}^{i\vartheta}$ accounting for the polarization state of the impinging light. Under the approximation taken, the surface current density reduces to

$$\mathbf{J}(t) = \frac{ev_{\mathrm{F}}}{\pi^2}\int(\cos\phi\hat{\mathbf{y}} - \sin\phi\hat{\mathbf{x}})\left\{[\Gamma_{\mathbf{k}}^{(1-)} - \Gamma_{\mathbf{k}}^{(1+)*}]\mathrm{e}^{-i\omega t} + [\Gamma_{\mathbf{k}}^{(3-)} - \Gamma_{\mathbf{k}}^{(3+)*}]\mathrm{e}^{-3i\omega t} + [\Gamma_{\mathbf{k}}^{(5-)} - \Gamma_{\mathbf{k}}^{(5+)*}]\mathrm{e}^{-5i\omega t} + \mathrm{c.c.}\right\} d^2\mathbf{k}. \quad (9a)$$

As we have discussed in the main paper, in our simulations we observe a drastic drop of the third and fifth harmonic parts of the current for circularly polarized light, while the fundamental harmonic remains unaffected. In order to understand the vanishing harmonic generation for circularly polarized light, we examine the equation above. The third and fifth harmonic currents are given by

$$\mathbf{J}_{3\omega}(t) = \mathrm{Re}\left\{\left[\int(\cos\phi\hat{\mathbf{y}} - \sin\phi\hat{\mathbf{x}})(\eta_{\mathbf{k}}^*)^3 f\left(|\eta_{\mathbf{k}}|^2\right) d\phi\right]\mathrm{e}^{-3i\omega t}\right\}, \quad (10a)$$

$$\mathbf{J}_{5\omega}(t) = \mathrm{Re}\left\{\left[\int(\cos\phi\hat{\mathbf{y}} - \sin\phi\hat{\mathbf{x}})(\eta_{\mathbf{k}}^*)^5 g\left(|\eta_{\mathbf{k}}|^2\right) d\phi\right]\mathrm{e}^{-5i\omega t}\right\}, \quad (10b)$$

where f and g are involved functions of $|\eta_{\mathbf{k}}|^2$, and $\eta_{\mathbf{k}} = \sin\phi - \cos\phi\mathrm{e}^{i\vartheta}$. For linearly polarized light ($\vartheta = 0$) $|\eta_{\mathbf{k}}|^2$ depends on ϕ, and the integrals above show a complicated dependence on ϕ resulting in non vanishing third and fifth harmonic currents. On the contrary, for circularly polarized light ($\vartheta = \pm\pi/2$) $|\eta_{\mathbf{k}}|^2 = 1$ and is thus independent on ϕ. Thus, the above integrals reduce to

$$\mathbf{J}_{3\omega}(t) = \mathrm{Re}\left\{\pm i\left[\int(\sin\phi\hat{\mathbf{x}} - \cos\phi\hat{\mathbf{y}})\mathrm{e}^{\mp 3i\phi}d\phi\right] f(1)\mathrm{e}^{-3i\omega t}\right\} = 0, \quad (11a)$$

$$\mathbf{J}_{5\omega}(t) = \mathrm{Re}\left\{\pm i\left[\int(\cos\phi\hat{\mathbf{y}} - \sin\phi\hat{\mathbf{x}})\mathrm{e}^{\mp 5i\phi}d\phi\right] g(1)\mathrm{e}^{-5i\omega t}\right\} = 0. \quad (11b)$$

As we discuss in the main paper, the physical origin of this cancellation stems from the phase mismatch of electron trajectories from opposite points in the reciprocal space [see Fig. S1]. In Fig. S2 we contour plot the dependence of the complex current amplitudes amplitudes j_x^{ω}, $j_x^{3\omega}$, and $j_x^{5\omega}$ over the dimensionless electron wavenumber $2v_{\mathrm{F}}\mathbf{k}/\omega$ for wavelength $\lambda = 2\pi c/\omega = 3.1\ \mu$m, impinging intensity $I_0 = 10$ GW/cm^2, and several polarization states ϑ, where we have defined such amplitudes as $\mathbf{J}_{\omega}(t) = \mathrm{Re}[\int \mathbf{j}^{\omega}(\mathbf{k})d^2\mathbf{k}\mathrm{e}^{-i\omega t}]$, $\mathbf{J}_{3\omega}(t) = \mathrm{Re}[\int \mathbf{j}^{3\omega}(\mathbf{k})d^2\mathbf{k}\mathrm{e}^{-3i\omega t}]$, and $\mathbf{J}_{5\omega}(t) = \mathrm{Re}[\int \mathbf{j}^{5\omega}(\mathbf{k})d^2\mathbf{k}\mathrm{e}^{-5i\omega t}]$. The y components of the current amplitudes j_y^{ω}, $j_y^{3\omega}$, and $j_y^{5\omega}$ exhibit a similar behavior.

Supplementary Note 3: Atomistic quantum-mechanical simulations

We consider a graphene ribbon containing $M \to \infty$ unit cells with period b along its direction of translational symmetry. Following a previously reported procedure[5–7], we construct the corresponding one-electron

wave functions from a tight-binding Hamiltonian $H_{\rm TB}$ (assuming a nearest-neighbor hopping energy of 2.8 eV) as $|j,k\rangle = \sum_{l,m} a_{jl,k} {\rm e}^{ikmb} |l,m\rangle / \sqrt{M}$, where j denotes the band index, k is the in-plane Bloch wave vector along the ribbon, $|l,m\rangle$ is the 2p carbon orbital at site $\mathbf{R}_l$ in unit cell m, and $a_{jl,k}$ are complex expansion coefficients. The optical response is simulated via direct numerical integration of the single-electron density matrix equation of motion,

$$\frac{\partial \rho}{\partial t} = -\frac{i}{\hbar}[H_{\rm TB} - e\phi, \rho] - \frac{1}{2\tau}\left(\rho - \rho^0\right), \tag{12}$$

where $\phi = -\mathbf{R}_l \cdot \mathbf{E}(t) - 2e\sum_{l',m'} v_{ll',mm'} \rho_{l'l',m'm'}$ is the self-consistent electric potential, with $v_{ll',mm'}$ denoting the Coulomb interaction between atom l in unit cell m and atom l' in unit cell m'. Relaxation in Supplementary Equation (12) brings us back to the equilibrium density matrix ρ^0 at a rate τ^{-1}. In the state representation, the relaxed matrix elements $\rho^0_{jj',kk'} = f_{j,k}\delta_{jj'}\delta_{kk'}$ are constructed by populating electron states according to the Fermi-Dirac distribution (see occupation numbers $f_{j,k}$). Here we consider light impinging along the graphene plane normal, with polarization $\mathbf{E}_0$ directed across the ribbon [i.e., the incident light field is $\mathbf{E}(t) = {\rm Re}\left\{\mathbf{E}_0 \exp(-i\omega t)\right\}$]. Upon integration of Supplementary Equation (12), the time-dependent elements ρ_{ll} yield the induced dipole moment per unit length of the nanoribbon

$$p(t) = -2e\sum_l \mathbf{R}_l \cdot \mathbf{E}_0 \int_{-\pi/b}^{\pi/b} \frac{dk}{2\pi}\left(\rho_{ll,kk} - \rho^0_{ll,kk}\right). \tag{13}$$

Supplementary References

[1] Ishikawa, K. L. *Phys. Rev. B* **82**, 201402(R) (2010).
[2] Ishikawa, K. L. *New J. of Phys.* **15**, 055021 (2013).
[3] Sipe, J. E. & Ghahramani, E. *Phys. Rev. B* **48** 11705 (1993).
[4] Marini, A., Cox, J.D. & García de Abajo, F. J. *Phys. Rev. B* **95**, 125408 (2017).
[5] Thongrattanasiri, S., Manjavacas, A. & García de Abajo, F. J. *ACS Nano* **6**, 1766 (2012).
[6] Cox, J. D. & García de Abajo, F. J. *Nat. Commun.* **5**, 5725 (2014).
[7] Cox, J. D., Silveiro, I. & García de Abajo, F. J. *ACS Nano* **10**, 1995 (2015).
